# Genome Instability Caused by a Germline Mutation in the Human DNA Repair Gene *POLB*

**DOI:** 10.1371/journal.pgen.1003086

**Published:** 2012-11-08

**Authors:** Robert W. Sobol

**Affiliations:** 1 Department of Pharmacology and Chemical Biology, University of Pittsburgh School of Medicine, Pittsburgh, Pennsylvania, United States of America; 2 University of Pittsburgh Cancer Institute, Hillman Cancer Center, Pittsburgh, Pennsylvania, United States of America; 3 Department of Human Genetics, University of Pittsburgh Graduate School of Public Health, Pittsburgh, Pennsylvania, United States of America; Baylor College of Medicine, United States of America

DNA polymerase ß (Polß) is recognized as an essential DNA repair protein [1], [2]. Although the smallest of the human DNA polymerases [3], [4], this 335–amino-acid protein is the primary DNA polymerase in the base excision repair (BER) pathway [5]. A majority of the 20,000 DNA lesions per day that each human cell is faced with are repaired by the BER pathway [6]. These include products of base depurination and depyrimidination (abasic sites), deamination of cytosine and 5-methylcytosine, oxidation products such as 8-oxo-7,8-dihydro-2′-deoxyguanosine (8-oxodG), thymine glycol and lipid peroxidation products, as well as methylation modifications such as N7-guanine [7], [8]. Failure to repair these spontaneous or endogenously induced DNA base lesions as well as the numerous base modifications that arise from environmental or exogenous sources can result in multiple cellular effects, including cell death, gene mutations, gene rearrangements, and/or decreased cell growth rate. Polß facilitates the repair of these base lesions in concert with different proteins of the BER pathway depending on the lesion [2], [7]. Once the base lesion is removed by one of 11 DNA glycosylase enzymes and the resulting abasic site is hydrolysed by the endonuclease APE1, Polß is recruited to the lesion via an interaction with the BER scaffold protein XRCC1 [9], [10] and the DNA damage sensor PARP1 [11]–[13]. Polß then conducts two essential enzymatic functions: 5′dRP lyase–mediated gap tailoring and DNA polymerase–mediated DNA synthesis to fill the gap [2], [3]. The 5′dRP lyase activity functions to “tailor” the gap by removing the sugar-phosphate residue that remains after APE1 cleaves the DNA backbone, and then the polymerase activity adds the newly synthesized nucleotide that was removed during repair. Considering the critical and essential role of these two enzymatic activities, the important protein–protein interactions between Polß and several BER proteins [14], as well as the increasing number of post-translational modifications suggested to affect Polß function and stability [15], it may not be surprising that a significant number of somatic mutations in *POLB* have been observed in cancer (Table 1). Within the 33 Kb *POLB* gene (PubMed geneID #5423), as many as 567 SNPs have been identified (see dbSNP). However, only 34 SNPs are in or near the coding region (22 are found in exons), and only two have been confirmed in larger cohorts. These two germline *POLB* mutants (R137Q; rs12678588 and P242R; rs3136797) have been reported to be present in as much as 0.6% and 2.4% of the human population, respectively [16], [17]. However, little is known about the functional impact that results from these single amino acid alterations. An earlier study on the Polß (R137Q) mutant (rs12678588) suggested that the R137Q mutation impairs function of the purified protein. Further, when produced in mouse cells, the R137Q mutant protein interfered with Polß binding to PCNA [18] and the response of mouse cells to DNA-damaging agents, although no information was provided on the impact of this mutation on genome stability. Whereas the Polß (P242R) mutant allele (rs3136797) has been linked with altered incidence of cancer in several studies [19]–[21], there have been few or no studies defining the impact of this SNP on Polß function, DNA repair capacity, and genome maintenance in human cells.

**Table 1:** Germline and somatic *POLB* mutants.*

WT Residue	Residue Number	Mutant Residue	Polß Domain	Functional or Correlative Effect of Mutation	Citation
Gln	8	Arg	8K	*n.d.*	[17]
Leu	22	Pro	8K	Loss of 5′dRP lyase activity	[37]
Leu	22	Pro	8K	Suppressed BER activity	[38]
Lys	27	Asn	8K	Decreased catalytic (5′dRP lyase) activity	[39]
Tyr	39	Cys	8K	*n.d.*	[38]
Gly	80	Arg	8K	*n.d.*	[40]
Ile	88	Val	8K	*n.d.*	[41], [42]
Phe	114	Ser	Fingers	*n.d.*	[41]
Gly	118	Glu	Fingers	*n.d.*	[41]
Glu	123	Lys	Fingers	Decreased catalytic (polymerase) activity	[39]
Arg	137	Gln	Fingers	Decreased interaction with PCNA, reduced polymerase activity	[18]
Arg	137	Gln	Fingers	*n.d.*	[17]
Arg	137	Gln	Fingers	Haplotype analysis	[16]
Asp	160	Asn	Palm	*n.d.*	[38]
Asp	160	Asn	Palm	Increase in cellular transformation	[27]
Lys	167	Ile	Palm	*n.d.*	[41]
Gly	179	Arg	Palm	*n.d.*	[41]
Arg	182	Gly	Palm	*n.d.*	[43]
Arg	183	Gly	Palm	*n.d.*	[41]
Glu	186	Gly	Palm	*n.d.*	[41], [42]
Glu	216	Lys	Palm	No observed change in activity	[39]
Gly	231	Asp	Palm	Decreased catalytic rate and decreased binding affinity of nucleotides	[44]
Glu	232	Lys	Palm	Decreased catalytic (polymerase) activity	[39]
Met	236	Leu	Palm	Decreased catalytic (polymerase) activity	[39]
Cys	239	Arg	Palm	*n.d.*	[38]
Pro	242	Arg	Palm	*n.d.*	[17], [45]
Pro	242	Arg	Palm	Decreased catalytic (polymerase) activity	[39]
Pro	242	Arg	Palm	Haplotype analysis	[16]
Pro	242	Arg	Palm	Decreased risk of colorectal cancer	[19]
Pro	242	Arg	Palm	Increase in cellular transformation and genome instability	[22]
Ile	260	Met	Palm	Misalignment-mediated errors in dipyrimidine sequences	[46]
Ile	260	Met	Palm	Increase in cellular transformation	[28]
Tyr	265	Cys	Thumb	Increase in mutation frequency	[23], [24]
Tyr	265	Cys	Thumb	Increase in BER intermediates, chromosome aberrations, and DNA breaks	[47]
Asn	281	Ser	Thumb	*n.d.*	[40]
Glu	288	Lys	Thumb	Increase in mutations at A/T base pairs	[48]
Lys	289	Met	Thumb	Increase in mutation frequency	[49]
Lys	289	Met	Thumb	*n.d.*	[45], [50]
Lys	289	Met	Thumb	Increase in cellular transformation	[28]
Asn	294	Asp	Thumb	*n.d.*	[38]
Glu	295	Lys	Thumb	Decreased polymerase activity, acts as a dominant negative	[51]
Glu	295	Lys	Thumb	Loss of BER and DNA polymerase activity	[38]
Glu	295	Lys	Thumb	Decreased polymerase activity that may stem from steric interaction with Arg258	[52]

*n.d.* = not determined.

* See [53] for an extensive list of *POLB* gene mutations recently identified in colorectal tumors.

In this issue of *PLOS Genetics*, Sweasy and colleagues conducted a detailed analysis of the *POLB* germline–coding SNP rs3136797 [22]. This polymorphism alters amino acid 242, changing the amino acid from a proline (P; Pro) to an arginine (R; Arg). To determine whether the P242R mutation affected genome stability in human or mouse cells, the wild-type (WT) or P242R protein was produced in human normal mammary epithelial cells (MCF10A) and in mouse embryonic fibroblasts (MEFs). In both cell lines, the synthesis of the P242R protein led to an increase in genomic alterations. Analysis of metaphase spreads showed that the P242R protein induced an increase in chromosome breaks and a significant increase in fragmented chromosomes and chromosome fusions. In other reports, cancer-specific mutations in Polß (e.g., Y265C) induced an increase in mutant frequency [23], [24] that could explain the increase in chromosome alterations seen with P242R. However, cells producing the P242R protein were found to have the same mutant frequency as those expressing WT Polß. The lack of an increase in mutations together with the increase in chromosomal instability suggested that the Polß (P242R) protein may promote the accumulation of DNA strand breaks during repair. To test this hypothesis, the cells were treated with methyl methanesulfonate (MMS) to induce DNA damage repaired by Polß [5]. As suspected, exposure of the cells expressing the P242R mutant to MMS induced a greater level of single-strand and double-strand DNA breaks. The increase in single-strand breaks and related BER intermediates was measured by the alkaline Comet assay [25], and an increase in the number of DNA double-strand breaks was indirectly determined by measuring an increase in γ-H2AX foci [26]. A second phenotype that Sweasy and colleagues have linked with cancer mutants of Polß is the ability to induce cellular transformation, as was seen with the D160N, I260M, and K289M Polß mutants [27], [28]. Similarly, production of the P242R mutant protein in mouse cells (C127λ) or human cells (MCF10A) increased growth in soft agar significantly in comparison with expression of WT Polß.

Yamtich et al. [22] then used both cellular analysis and biochemical measurements to evaluate the functional impact of the P242R mutation. WT MEF cells (expressing endogenous WT Polß) were engineered to produce either WT human Polß or the P242R mutant protein and were then exposed to MMS to measure cellular survival. Both WT- and P242R-expressing cells responded equally except at the highest doses of MMS. Next, Polß-knock out (KO) MEFs engineered to express either WT human Polß or the P242R mutant were exposed to MMS to determine whether the P242R mutant could restore (complement) resistance to MMS. In this case, there was a small but significant difference in response, suggesting that the P242R mutant was mildly defective in BER. A strength of the Sweasy lab's study is the use of cell biology analyses as well as detailed biochemical evaluation of these mutant proteins. Yamtich et al. [22] expressed and purified the WT and P242R mutant proteins from *E. coli* and measured the rate of DNA polymerase activity by two separate kinetic analyses. This provided the opportunity to determine whether the decreased BER capacity observed in the Polß-KO MEFs expressing the P242R mutant in response to MMS and the increase in DNA breaks were the result of a defect in the polymerase activity of the P242R mutant. In both cases, they found that the mutation (P242R) caused a decrease in the rate of DNA polymerase activity. However, the protein bound to the DNA substrate with affinity equal to that of the WT enzyme. The slow polymerase activity of the P242R protein therefore is likely to promote the accumulation of BER intermediates, inducing genome alterations when the cell is exposed to DNA-damaging agents [29].

Defects in Polß can have significant cellular ramifications, especially in response to DNA-damaging agents that require Polß and BER for repair. Complete loss of Polß function can trigger an increase in cell death in response to high doses of genotoxins [5], [30] and an increase in genome alterations even at low doses [29]. Additional cellular responses to DNA damage when Polß is defective may include PARP1 activation and alterations in bioenergetic metabolites such as NAD^+^ [31]. The steady-state expression level of Polß is also reported to be regulated by the proteasome via ubiquitylation [32], suggesting that some Polß mutants may have altered stability. In this regard, the observation that the P242R mutant protein has a functional defect now opens the door for further studies to clarify the mechanisms and cellular impacts of other defects in Polß. It has been suggested that tumor-specific defects in BER, such as a defect in Polß, may be exploited for selective therapeutic options [33]. Cells producing the mutant protein (P242R) have a higher level of DNA strand breaks and increased cellular transformation, and so it is possible that the Polß (P242R) protein may be considered a driver of cancer formation. It remains to be determined whether the presence of this mutant protein (P242R) provides therapeutic selectivity.

**Figure 1:**
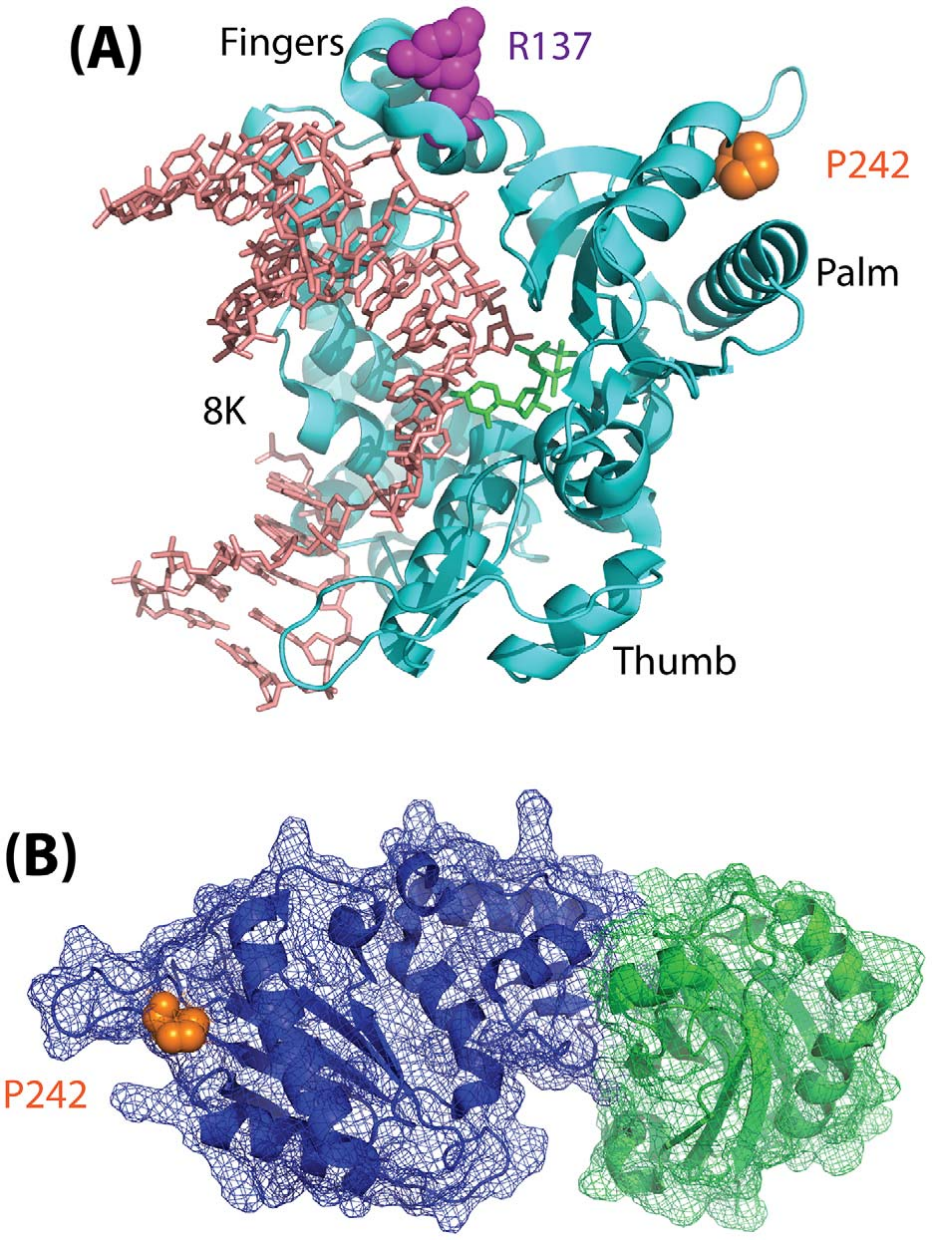
Model depicting the structure of Polß. (A) Structure (pdb2fms) depicting DNA Polymerase ß (Polß) with a gapped DNA substrate and dUMPNPP with magnesium in the catalytic site [35]. The image is a cartoon rendition of the polypeptide chain of Polß in teal, the gapped DNA substrate in salmon, and the incoming dUMPNPP base in green. Amino acids known to be altered by germline mutations are shown using a space-filling rendering: R137 (magenta) and P242 (orange). The fingers, palm, and thumb domains of Polß are indicated. The 8K domain is at the back of the structure, facing away from the plane of the image, and is shown behind the DNA in this orientation. (B) Structure (pdb3lqc) depicting oxidized XRCC1 bound to the Polß palm/thumb domains [36]. The image is a cartoon rendition of the palm and thumb domains of Polß in blue, with a mesh illustrating the surface of the structure (amino acids 150–335), and a cartoon rendition of the oxidized form of XRCC1 in green, with a mesh illustrating the surface of the structure (amino acids 1–151). Amino acid P242 (orange) is shown using a space-filling rendering. The images were generated using PyMOL (Molecular Graphics System, Version 1.2r3pre; Schrödinger, LLC; http://pymol.org/).

Finally, it remains to be determined how a mutation (P242R) so distant from the Polß active site, and which does not interfere with binding to XRCC1 (Figure 1), can have such a significant effect on the function of Polß. Given the subtle yet significant impact of the Polß (P242R) mutant on cellular function and genome stability in response to DNA damage as described by Yamtich et al [22], further analysis of this mutant protein is warranted. The P242 amino acid is located in a loop domain that is essential for enzymatic activity [34], so it is likely that the alteration of the amino acid from P to R changes the movement of the loop and may also change the overall architecture of the protein. To more completely appreciate the subtle yet significant defect associated with this germline mutation, it is therefore suggested that future studies be conducted to determine the structure of the ternary complex of Polß (P242R) with DNA and an incoming nucleotide. In addition, whole animal studies should be considered so as to determine whether the genome instability and cellular transformation results described [22] extend to additional cell types. As a germline mutation, analysis of the P242R mutant protein in an animal model will provide valuable insight into the possible effects on human health.
